# Suppressive GLI2 fragment enhances liver metastasis in colorectal cancer

**DOI:** 10.18632/oncotarget.28170

**Published:** 2022-01-15

**Authors:** Ruiko Ogata, Shiori Mori, Hitoshi Ohmori, Shingo Kishi, Rina Fujiwara-Tani, Takamitsu Sasaki, Yukiko Nishiguchi, Chie Nakashima, Kei Goto, Isao Kawahara, Yi Luo, Hiroki Kuniyasu

**Affiliations:** ^1^Department of Molecular Pathology, Nara Medical University, Kashihara, Nara 634-8521, Japan; ^2^Key Laboratory of Neuroregeneration of Jiangsu and Ministry of Education, Co-Innovation Center of Neuroregeneration, Nantong University, Nantong, Jiangsu Province 226001, China

**Keywords:** linoleic acid, stemness, GLI2, ubiquitination, colorectal cancer

## Abstract

Linoleic acid (LA) has been shown to cause inflammation and promote development of colorectal cancer (CRC). Moreover, many literatures show that LA is associated with cancer metastasis. Metastatic cancer cells have high stemness, suggesting that LA might affect the stemness of cancer cells. In this study, we examined the effect of LA on the hedgehog system, which affects cancer stemness. In CT26 cells, LA treatment induced the expression of sonic hedgehog *(Shh)*; the signal transduction factor, and glioma-associated oncogene homolog *(Gli)* *2*, whereas the expression of SRY-box transcription factor *(Sox)* *17* was suppressed. Furthermore, LA reduced GLI2 ubiquitination, resulting in an increase in the N-terminal fragment of GLI2, known as suppressive GLI2, produced by cleavage of GLI2. LA-induced cleaved GLI2 was also detected in Colo320 and HT29 human CRC cells. Knocking down *Gli2* abrogated the LA-mediated suppression of *Sox17* expression. These results suggest that LA promotes tumor cell stemness by increasing of suppressive GLI2 fragments via GLI2 modification. In mouse liver metastasis models, LA enhanced metastasis with production of the suppressive GLI2 fragments in CT26 and HT29 cells, whereas knockdown of *GLI2* abrogated LA-induced metastatic activity. In human CRCs, the cases with liver metastasis showed the suppressive GLI2 fragments. This study provides mechanistic insights into LA-induced stemness in colon cancer cells. This finding suggests that dietary intake of LA might increase the stemness of cancer cells and enhance metastatic activity of the cancer.

## INTRODUCTION

The incidence of colorectal cancer (CRC), a leading cause of cancer-related deaths worldwide, has recently increased [1]. In the United States, nearly 150,000 cases of colorectal cancer and more than 50,000 deaths were reported in 2020 [1]. Many causes of CRC are related to lifestyle; Western eating habits, overnutrition, and a lack of exercise are known risk factors for colorectal cancer. In particular, colon carcinogenesis is closely related to diet. It is widely known that inflammation caused by the metabolic processing of fatty acids (generated by lipid overdose, alcohol abuse, and overconsumption of processed meat) increases the risk of tumor progression [2].

In previous studies, linoleic acid enhances metastasis of experimental gastrointestinal cancer [3, 4]. We have reported that LA enhances the growth, survival, invasion, and metastasis of cancer cells by promoting stemness [5–7]. LA also breast cancer metastasis via eicosanoid biosynthesis and protein kinase C activation [8]. The expression of the long-chain fatty acid receptor, G protein-coupled receptor 40, is increased in patients with CRC who have high blood triglyceride levels, and it is associated with an increased risk of cancer progression [9]. However, the mechanism that LA enhances cancer stemness is still unclear.

Since it has been reported that sonic hedgehog (SHH) increases cancer stemness [10]. The activation of SHH signaling is also a feature of cancer stem cells [11]. In the gastrointestinal tract, SHH is characteristically expressed in the stomach and large intestine [12]. SHH is overexpressed in CRCs [13], and activation of SHH signaling correlates with the metastatic potential of CRCs [14]. The hedgehog (Hh) pathway involves signaling via one of the three Hh ligands (SHH, Indian HH [IHH], and Desert HH [DHH]) to the glioma-associated oncogene (GLI) transcription factors [15]. This process is mediated by interplay between the inhibitory receptor, patched (PTCH1), and the activating receptor, smoothened (SMO); the process is also influenced by protein kinase A (PKA), and cyclic adenosine monophosphate (cAMP). The signal-activated form of GLI migrates to the nucleus and binds to the promoter, stimulating the transcription of Hh target genes [16]. Interestingly, full-length GLI2 promotes gene expression, whereas the N-terminal cleavage product of GLI2 is known to repress gene expression [17]. We also paid attention to this GLI2 cleavage product in this study.

Although LA exerts various effects on carcinogenesis and cancer progression, the mechanism through which it enhances stemness is still unclear. Therefore, in this study, we investigated the mechanisms underlying LA-mediated stemness, with an emphasis on investigating the potential role of SHH signaling.

## RESULTS

### Effect of LA on expression of hedgehog-associated proteins

**Figure 1:**
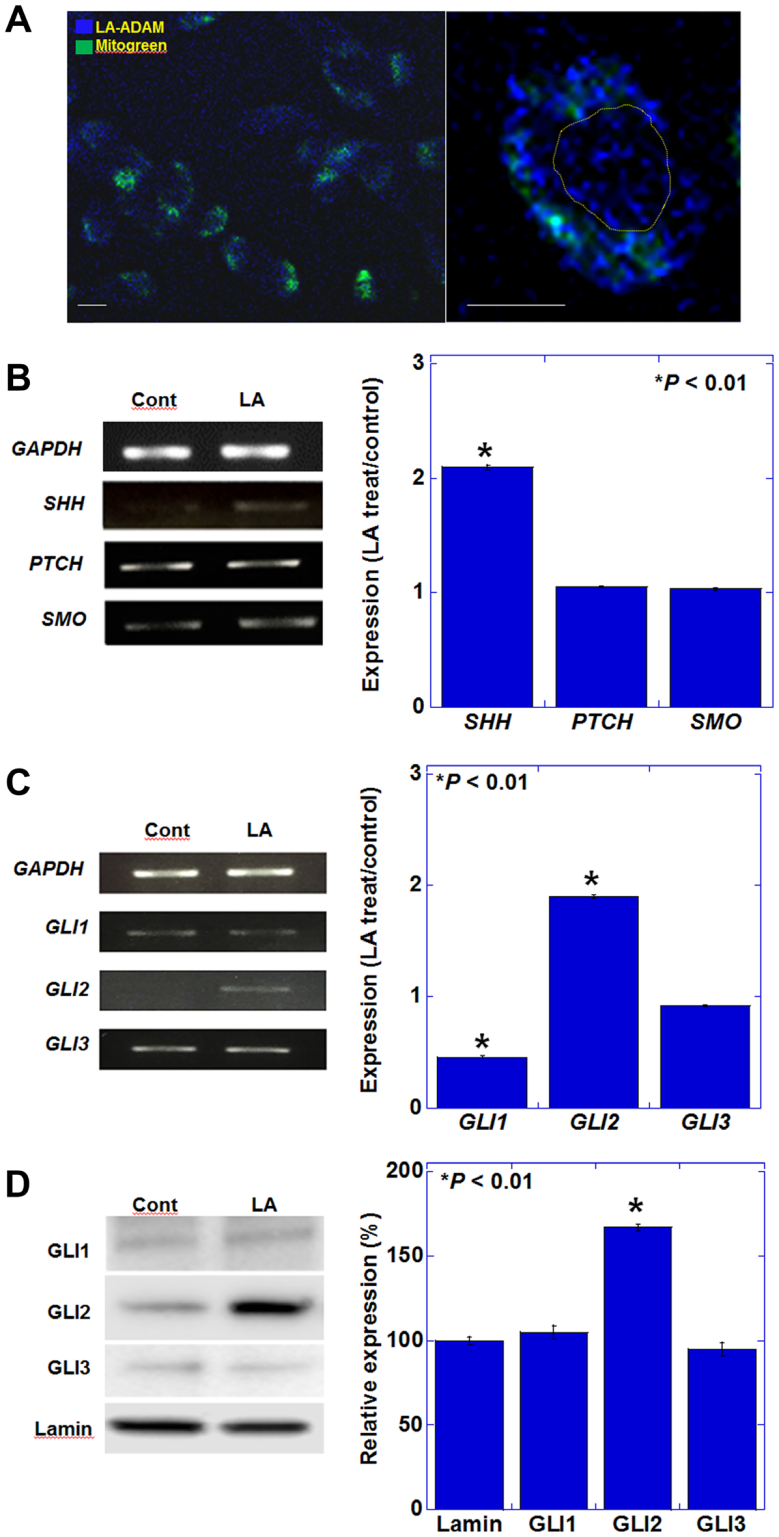
Effect of LA on the expression of genes encoding SHH pathway mediators in CT26 cells. (**A**) Intracellular localization of ADAM-labeled LA. Mitochondria were labeled with MitoGreen. Right panel showed a single cell. Yellow circle indicates nucleus. Scale bar, 20 μm. (**B**) Gene expression of hedgehog ligand and receptors examined by RT-PCR. (**C**) Gene expression of hedgehog signaling pathway. (B, C) The graphs represent the results of quantitative RT-PCR. (**D**) Protein levels in nuclear fraction of hedgehog signaling pathway. The graphs represent semi-quantification of the results shown on the left. (A-D) CT26 cells were treated with 35 μM of LA, which was equivalent to inhibitory concentration 25. Error bars indicate standard deviations based on three independent experiments. Asterisks indicate statistical significance. Statistical differences were calculated by ordinary analysis of variance. Abbreviations: ADAM: 9-anthryldiazomethane; LA: linoleic acid; GAPDH: glyceraldehyde 3-phosphate dehydrogenase; SHH: sonic hedgehog; PTCH: patched; SMO: smoothened; GLI: glioma-associated oncogene homolog.

We first examined the intracellular localization of LA in CT26 mouse colon cancer cells using fluorescently labeled LA. LA accumulation was observed in the cytoplasm and nucleus. (Figure 1A). The cytosolic signals were partially colocalized with mitochondria, suggesting that fatty acids are involved in processes by β-oxidation. In contrast to the extracellular LA concentration (35 μg/g), the fluorescence intensities of whole cell lysate and nuclear fraction showed a 9.4-fold and 1.8-fold, respectively, increase in LA accumulation per g/h. It was suggested that LA might be involved in some nuclear signal. When we examined the expression of the SHH signaling-related genes, *Shh*, *Ptch*, and *Smo*, after LA treatment, we observed that only *Shh* exhibited increased expression (Figure 1B). GLIs are responsible for transmitting SHH signals to the nucleus and inducing gene expression [15]. The expression pattern of GLIs may be involved in the specific signal formation by LA. With respect to the GLI transcription factors, in LA-treated CT26 cells, the expression of *Gli2* increased, whereas *Gli1* expression was reduced (Figure 1C). To compare the activation levels of GLIs, we examined the nuclear protein levels of GLIs (Figure 1D). GLI2 protein in the nuclear fraction was increased; however, GLI1 and GLI3 was not increased.

### Effect of LA on promotion of stemness in CRC cells

**Figure 2:**
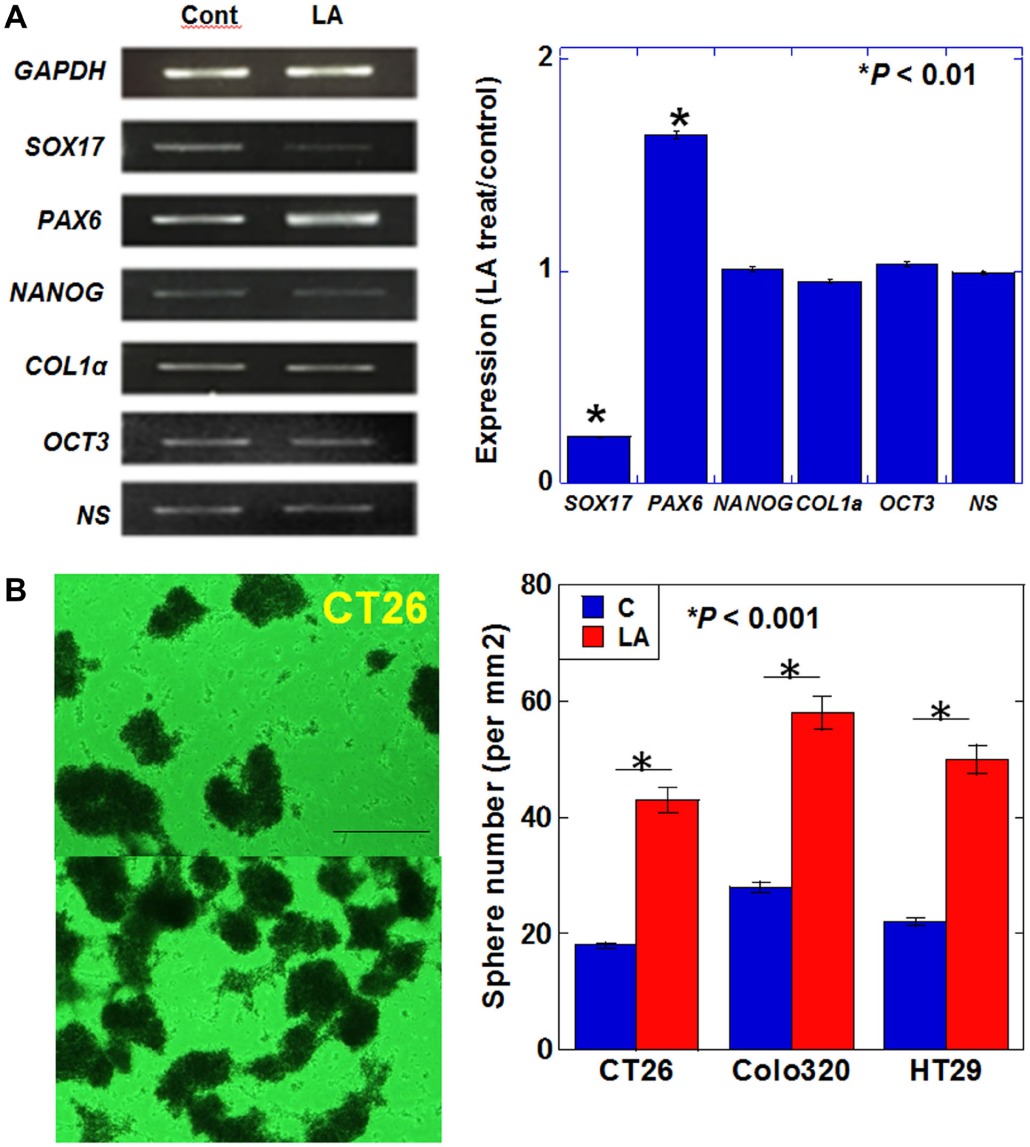
Effect of LA on stemness in CT26 cells. (**A**) Expression of stem cell-associated genes. (**B**) Sphere formation assay. The bar graph shows the quantification of total sphere numbers of 3 cell lines. Scale bar, 200 μm. (A, B) Cells were treated with 35 μM of LA, which was equivalent to inhibitory concentration 25. Error bars indicate standard deviations based on three independent experiments. Asterisks indicate statistical significance. Statistical differences were calculated by ordinary analysis of variance. Abbreviations: LA: linoleic acid; GAPDH: glyceraldehyde 3-phosphate dehydrogenase; SOX: SRY-box transcription factor; PAX: paired box; NANOG: nanog homeobox; COL: collagen; OCT: octamer-binding transcription factor; NS: nucleostemin.

It has been reported that SHH increases cancer stemness [10], and accordingly, we next examined the expression of genes related to cancer stemness in LA-treated CT26 cells. The results of reverse transcription-polymerase chain reaction analysis revealed a marked decrease in the expression of SRY-box transcription factor *(Sox) 17* and increased expression of paired box *(PAX) 6* (Figure 2A). We also observed that sphere formation (a characteristic of cancer stem cells) was enhanced by LA treatment in CT26, Colo320 and HT29 CRC cells (Figure 2B). These results suggest that LA treatment promotes stemness in CT26 cells and also Colo320 and HT29 cells.

### Effect of LA on SHH signaling

**Figure 3:**
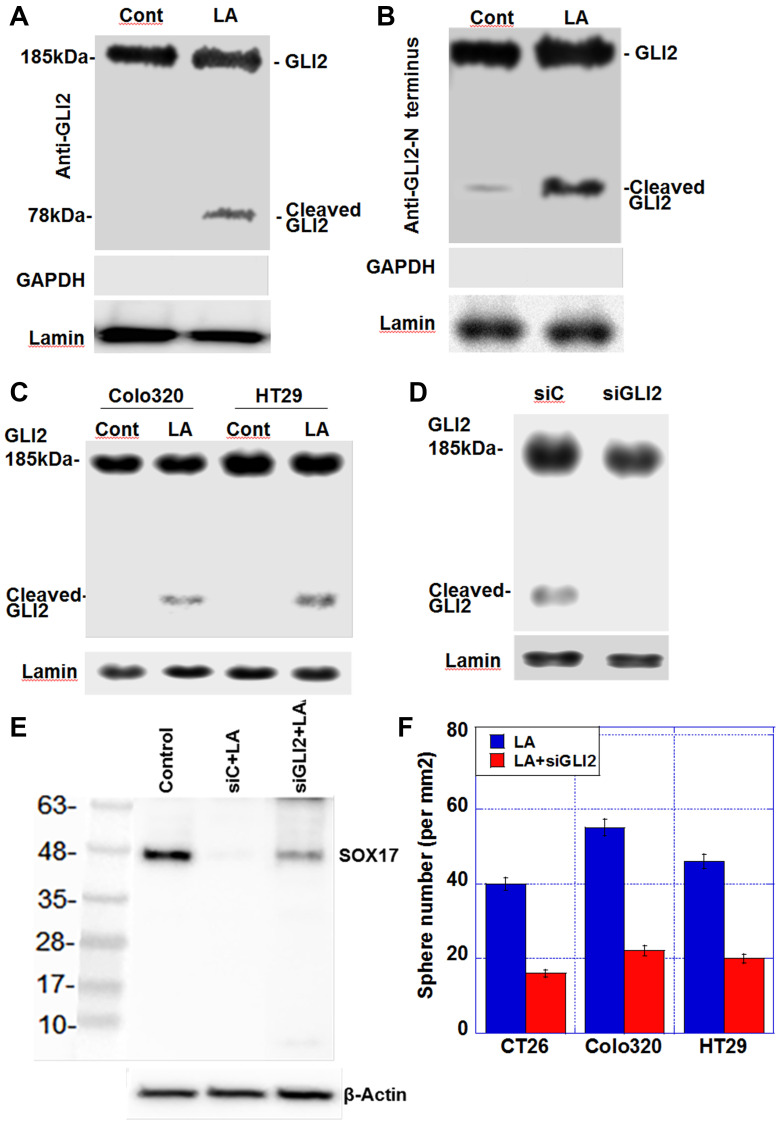
Effect of LA on generation of cleaved GLI2 in CT26 cells. (**A**) Nuclear protein levels of GLI2 and cleaved GLI2 in CT26 cells. (**B**) Nuclear protein levels of determined using an anti-GLI2 N-terminus antibody. GAPDH was examined as a cytosolic marker. (**C**) Nuclear protein levels of GLI2 and cleaved GLI2 in Colo320 and HT29 cells (**D**) Effect of siGLI2 on nuclear GLI2 protein levels in LA-treated CT26 cells. (**E**) Effect of *Gli2* knockdown on SOX17 expression in CT26 cells treated with LA. (**F**) Sphere formation assay. The bar graph shows the quantification of total sphere numbers of 3 cell lines. Production of SOX17 in untreated control, control siRNA-treated, and siGLI2-treated CRC cells. CRC cells were treated with 35 μM of LA, which was equivalent to inhibitory concentration 25. Abbreviations: LA: linoleic acid; GLI: glioma-associated oncogene homolog; GAPDH: glyceraldehyde 3-phosphate dehydrogenase; siGLI2: short interference RNA for GLI2; siC: short interference RNA; SOX: SRY-box transcription factor; Cont: control.

Figure 1 demonstrated that LA treatment induces changes in the expression of *Gli1* and *Gli2*, suggesting that they may be involved in the transcriptional regulation of genes, such as *Sox17*, in response to LA exposure. Next, we examined the nuclear levels of GLI2, which represents the active form of this transcription factor (Figure 3). The expression of full-length GLI2 (185 kDa) in the nucleus was present in LA-treated cells, but cleaved GLI2 (78 kDa) only demonstrated nuclear expression in response to LA treatment (Figure 3A).

The N-terminal portion of the cleaved GLI2 protein has been reported to possess repressive activity [18]. When we examined the nuclear protein samples from Figure 3A using an anti-GLI2 N-terminal antibody, we still detected a 78-kDa cleaved GLI2 fragment in LA-treated CT26 cells, indicating that the repressor form of the GLI2 protein was present in the nucleus (Figure 3B). 78-kDa cleaved GLI2 fragment in the nuclear fraction was also detected in LA-treated Colo320 and HT29 cells (Figure 3C). Knockdown of *Gli2* resulted in the loss of nuclear cleaved GLI2 expression in LA-treated CT26 (Figure 3D) and alleviated the LA-induced suppression of SOX17 expression (Figure 3E). Moreover, knockdown of *Gli2* abrogated enhanced sphere formation by LA (Figure 3F).

### Effect of LA on induction of GLI2 cleavage

**Figure 4:**
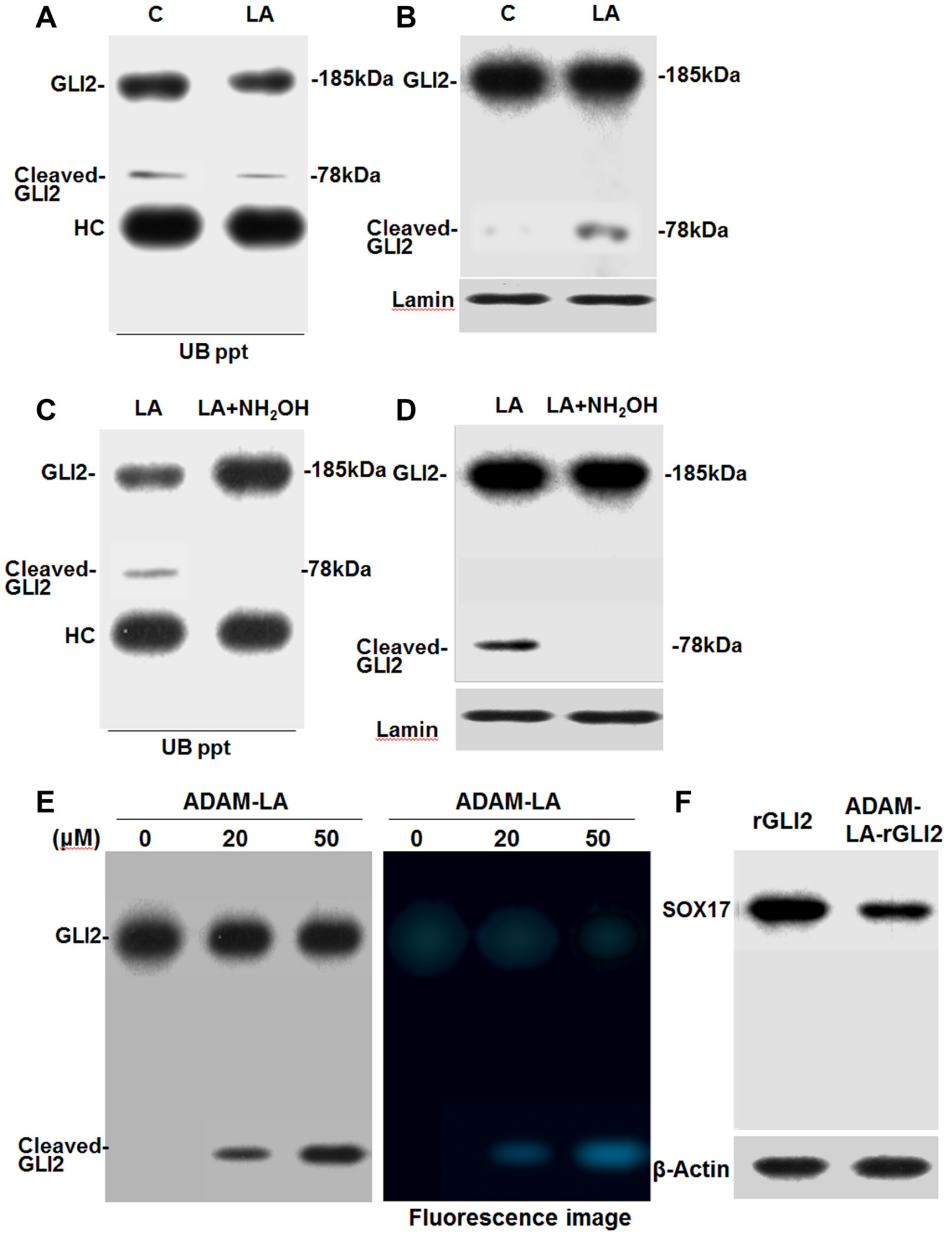
Effect of LA on the ubiquitination of GLI2 in CT26 cells. (**A**) Effect of LA on the ubiquitination of GLI2 in nuclear fraction of LA-treated CT26 cells. (**B**) The same nuclear extract of A was subjected to western blotting with anti-GLI2 antibody. (**C**) Effect of acylation inhibitor hydroxylamine (NH_2_OH, 1.75 mM) on LA-induced inhibition of GLI2 ubiquitination in nuclear fraction of CT26 cells. (**D**) The same nuclear extract of C was subjected to western blotting with anti-GLI2 antibody. (A, C) Nuclear fraction from LA- or LA+hydroxylamine-treated CT26 cells were immunoprecipitated using an anti-ubiquitin antibody (UB ppt). The electrophoresed UB ppt samples were detected by immunoblotting with an anti-GLI2 antibody. (**E**) LA-binding to recombinant GLI2. Mouse recombinant GLI2 (rGLI2) was incubated with ADAM-labeled LA (ADAM-LA). rGLI2 was transported to CT26 cells by Chariot^TM^. After 12 h culture, whole cell lysate was extracted from CT26 cells to detect GLI2 and cleaved GLI2 by western blot. In right panel, the membrane was also observed by excitation wavelength 365 nm, fluorescence wavelength 412 nm. (**F**) Using the whole cell lysate in G, SOX17 was detected. Error bars indicate standard deviations based on three independent experiments. Statistical differences were calculated by ordinary analysis of variance. Abbreviations: C: control; LA: linoleic acid; GLI: glioma-associated oncogene homolog; Cl-GLI2: cleaved GLI2; ADAM: 9-anthryldiazomethane.

GLI2 levels are regulated by ubiquitin-mediated degradation, which degrades C-terminal side of GLI2 (185 kDa) and generates N-terminal cleaved GLI2 fragments (78 kDa) [18, 19]. Therefore, we next examined whether LA treatment causes changes in the levels of ubiquitinated GLI2. Immunoprecipitation and western blot assay results showed that CT26 cells treated with LA exhibited reduced levels of ubiquitinated GLI2 and ubiquitinated cleaved GLI2 (Figure 4A). In contrast, total level of cleaved GLI2 was 11 times increased (Figure 4B). Co-treatment with hydroxylamine, an acylation inhibitor, prevented LA-induced decrease in ubiquitinated GLI2 levels (Figure 4C). Ubiquitinated cleaved GLI2 was disappeared. Notably, LA-induced cleaved GLI2 showed low level of ubiquitination. Moreover, hydroxylamine treatment abrogated LA-induced cleaved GLI2 increase (Figure 4D). To confirm the modifocation of GLI2, mouse recombinant GLI2 (rGLI2) incubated with ADAM-labeled LA was transported into CT26 cells (Figure 4E). The whole cell lysate from the CT26 cells, cleaved GLI2 was detected, which showed ADAM fluorescence. The cleaved band was thought to be derived from LA-conjugated rGLI2. As shown in Figure 4F, when we also detected SOX17 in the whole cell lysate, SOX17 was decreased by LA-conjugated rGLI2 treatment. Thus, cleaved GLI2 was thought to repress SOX17.

### Effect of LA diet on liver metastasis of CRC cells via cleaved GLI2 fragment in mouse models

**Figure 5:**
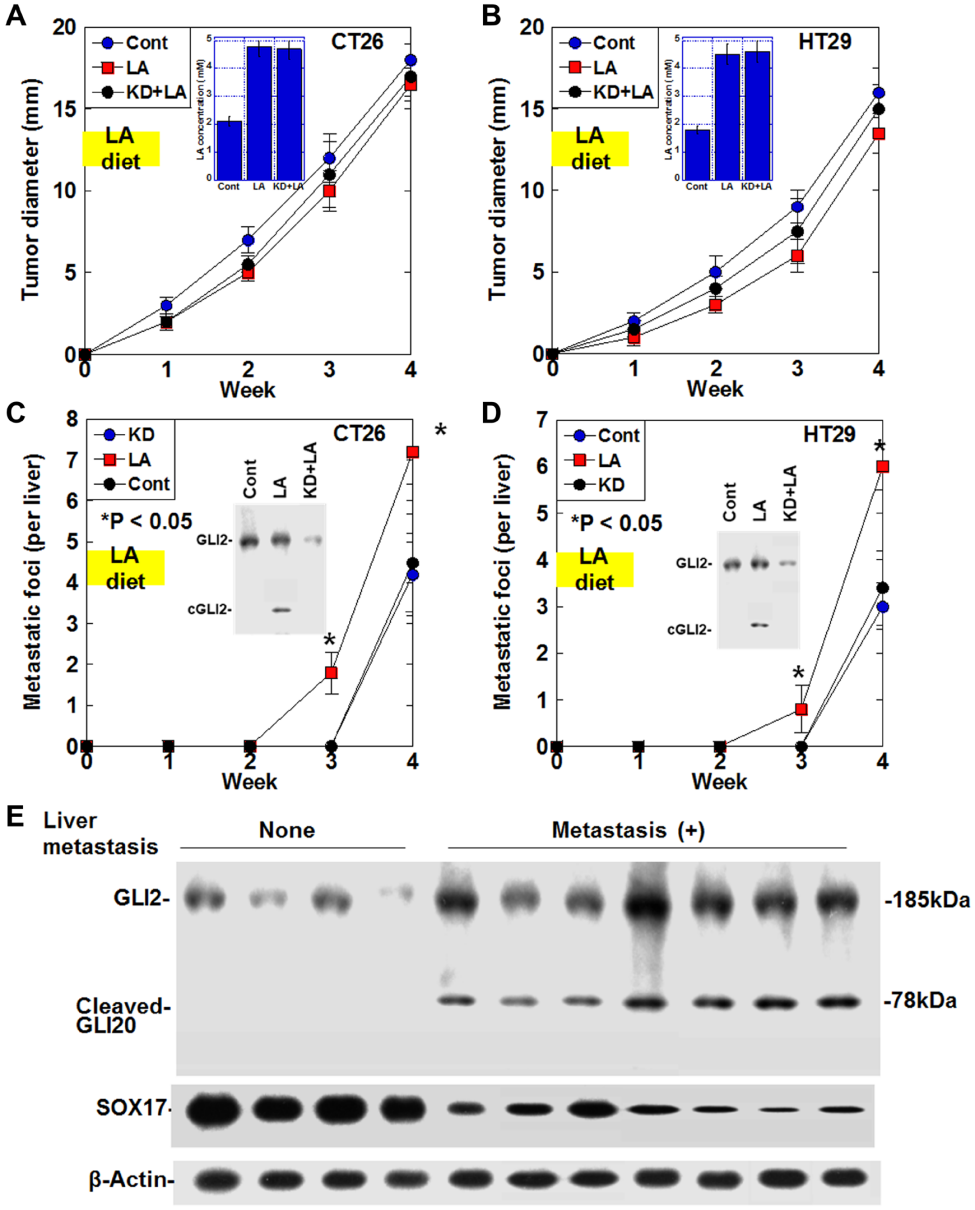
Effect of cleaved GLI2 fragment on liver metastasis in CRC. (**A**–**D**) Effect of LA on liver metastasis was examined in the orthotopic colon cancer models using CT26 cells-BALB/c mice and HT29 cells-nude mice. In GLI2 knockdown (KD) group, cells were knocked downed *GLI2* before inoculation. In LA or KD+LA groups, mice were fed with 15% LA diet for the first week. (A, B) Growth of the primary tumors in the cecum. (Insert) Plasma LA concentration. (C, D) Number of the liver metastasis foci. Error bars indicate standard deviations of 5 mice. Statistical differences were calculated by chi-square test. (Insert) Protein levels of GLI2 and the cleaved GLI2 fragment examined by immunoblotting. (**E**) Protein levels of GLI2, cleaved GLI2 and SOX17 were examined in the 11 CRCs with serosal invasion (pT3) and a few nodal metastasis (pN1) by immunoblotting. Abbreviations: LA: linoleic acid; GLI: glioma-associated oncogene homolog; cGLI2: cleaved GLI2; SOX: SRY-box transcription factor; Cont: control.

For analyzing the effect of LA-induced cleaved GLI2 fragment on cancer cells, mouse orthotopic colon cancer models were employed (Figure 5A–5D). Plasma LA levels increased 2.3-fold in mice fed the LA diet compared to mice fed the standard diet (Figure 5A, 5B). To the primary tumors in the cecum, LA or GLI2 knockdown did not affect their growth in both cell lines. Liver metastasis was enhanced in LA diet group, whose tumors showed cleaved GLI2 fragment in both cell lines (Figure 5C, 5D). In contrast, LA-enhanced liver metastasis was abrogated in GLI2 knockdown group, whose tumors showed disappeared cleaved GLI2 fragment in both cell lines.

### Effect of cleaved GLI2 fragment on liver metastasis in human CRCs

Finally, we examined cleaved GLI2 fragment in human CRC cases to evaluate the effect on liver metastasis (Figure 5E). In the 11 CRCs with serosal invasion (pT3) and 1–3 regional lymph node metastasis (pN1), the four CRCs without no liver metastasis showed no cleaved GLI2 fragment and high levels of SOX17. In contrast, all 7 cases with liver metastasis expressed cleaved GLI2 fragment and low levels of SOX17.

## DISCUSSION

In the present study, we examined the expression of stem cell markers in LA-treated CT26 cells and found that *Sox17* expression decreased, while *Pax6* expression increased. SOX17 is a transcription factor that enhances differentiation in the endoderm, and its expression is reportedly reduced in cancer stem cells [20, 21]. The transcription factor PAX6 is essential for self-renewal, pluripotency, and neurogenesis of neural stem cells in the central nervous system, including the cerebral cortex [22]. LA-induced downregulation of SOX17 expression and upregulation of PAX6 expression increased stemness. Therefore, the matching data generated in this study suggest that LA treatment can induce stemness in CT26 cells.

In our study, LA promoted the expression of GLI2 whereas GLI1 expression decreased. GLI1 promotes cell proliferation by inducing cyclin D1 expression [23], while GLI2 is known to increase cancer cell proliferation, tumorigenicity, and metastatic potential [24, 25]. Our data show that the generation of a cleaved GLI2 fragment further enhances stemness and metastability in CRC.

It is widely known that the cleaved products of GLI2, specifically the N-terminal segment, act as repressors [17]. Six serine residues from ^792^Ser distributed on the C-terminal side of the GLI2 cleavage site are phosphorylated by PKA or glycogen synthase kinase 3β. The β-transducin repeat containing protein/Skp1-cullin1-F-box ubiquitin ligase complex that binds to the serine phosphorylation site ubiquitinates lysine residues. GLI2 might undergo ubiquitination of clusters of lysine residues from ^773^Lys [26], and GLI2 is cleaved by degradation of this site [18, 27, 28].

So how does LA affect suppressive GLI2 activity? It is known that oxidized metabolites of LA, such as 13-hydroxyoctadecadienoic acid, can be transferred to the nucleus and become ligands for peroxisome proliferator-activated receptors [29]. Our results, in which fluorescently labeled LA showed a signal in the nucleus, suggest that LA is translocated into the nucleus. The results of our analysis revealed that LA treatment reduced the levels of ubiquitination of full-length GLI2 and cleaved GLI2. Inhibition of acylation abrogated LA-induced cleaved GLI2 production. These results suggested LA modified GLI2 on lysine residue by acylation. As fatty acids bind to lysine residues by acylation [30, 31], we hypothesized that LA-induced acylation of lysine residues in GLI2 might competitively inhibit ubiquitination at these sites. Protein lysine fatty acylation is a translational modification that has recently attracted attention. Fatty acids bind to lysine residues by enzymatic or non-enzymatic reactions [32]. Protein lysine fatty acylation regulates intracellular transport, intracellular localization, protein-protein and protein-lipid interactions [30]. In acylated full-length GLI2, reduced ubiquitination might decrease GLI2 cleavage. In contrast, in our data, the level of ubiquitination in cleaved GLI2 was significantly reduced by LA treatment, which suggests that high levels of lysine fatty acylation occurred in cleaved GLI2. Acylation of cleaved GLI2 might result in its stabilization and promotion of nuclear translocation. This suggests that LA promotes inhibitory regulation of gene expression by cleaved GLI2. A reporter assay should be performed to examine the effect on gene transcription by cleaved GLI2. However, in this study, nuclear translocation of GLI2 or cleaved GLI2 is used as an index of transcriptional activity, has been considered valid [33].

Our data indicate that LA treatment results in the increment of cleaved GLI2, which might regulate gene expression differently than full-length Gli2. To date, cleaved GLI2 has been reported to be a repressor [17, 28]. Downregulation of *Sox17* was the result of repression by cleaved GLI2. Interestingly, SOX17 downregulates PAX6 in embryonic stem cells [34, 35]. In LA-treated CT26 cells, SOX17 was downregulated by cleaved GLI2, and it is considered that the expression of PAX6, which had been repressed by SOX17, was enhanced. Additionally, our data showed LA treatment decreased *Gli1* expression and increased *Shh* expression. Since GLI2 upregulates *Gli1* expression [36], generation of suppressive GLI2 and decrease of full length GLI2 by LA provide repression of *Gli1*.

In the present study, the results of our analyses strongly suggested that GLI2 was suggested to be acylated in response to LA. Protein lysine fatty acylation is triggered by several enzymes including sirtuin [32]. Recently, non-enzymatic mechanisms of acylation by acetyl phosphate, acetyl CoA, and succinyl-CoA have also been reported [37]. Oxidative stress is also reportedly involved in LA-mediated acylation [38]. Although the mechanisms underlying GLI2 acylation were not elucidated in the current study, this will be an important focus for future research, including whether specific enzymes are required and whether other fatty acids facilitate GLI2 modification.

In this study, we found that LA generated an inhibitory fragment of GLI2, which repressed SOX17 and increased PAX6 expression, resulting in increased stemness. The effect of LA metabolism on the mechanism of LA may be important. In the future, it is necessary to consider the effects of LA on peroxide formation, prostaglandin formation, and integration into lipid bilayers. In this study, we identified SOX17 as a target gene of the GLI2 suppressive fragment, but it is necessary to extract the target genes by comprehensive analysis. In order to elucidate the repressive mechanism of the GLI2 suppressive fragment, it is necessary to introduce a vector expressing this fragment and analyze the promoter DNA sequence involved in the transcriptional activity using a luciferase assay. In addition, it is desirable to analyze the dietary intake of LA and the amount of GLI2 inhibitory fragments produced clinically.

The results from this study suggest that LA promotes the suppressive GLI2 via acylation, thereby promoting cancer stemness. We observed an increase in the nuclear levels of suppressive GLI2 fragments, which play a role in enhancing stemness. As a result, in the mouse model, LA promoted liver metastasis, which was abrogated by GLI2 knockdown. Furthermore, in human CRCs, an increase in cleaved GLI2 fragment and a corresponding decrease in SOX17 showed a strong association with liver metastasis. LA is considered a promoter of colorectal carcinogenesis, but our results indicate a novel link between LA and a malignant phenotype of CRCs via production of suppressive GLI2 fragment. These finding suggest that the LA-suppressive GLI2 fragment axis might be a relevant target for conquering metastasis of CRCs.

## MATERIALS AND METHODS

### Cells and reagents

The CT26 mouse colon cancer cell line was provided by Professor Isaiah J. Fidler (MD Anderson Cancer Center, Texas University, Houston, TX, USA). HT29 and Colo320 human carcinoma cell lines were purchased from Dainihon Pharmacy Co. (Tokyo, Japan). The cells were cultured in Dulbecco’s modified Eagle’s medium (Wako Pure Chemical, Osaka, Japan) supplemented with 10% fetal bovine serum (Sigma Chemical Co., St. Louis, MO, USA) at 37°C in a 5% CO_2_ atmosphere.

LA (Wako) was dissolved in 100% ethanol (20 μg/mL), and the same quantity of 100% ethanol was applied to control cells. CT26 cells were treated with 35 μM of LA, which was equivalent to inhibitory concentration 25.

### Sphere formation assay

Cells (1,000 cells per well) were seeded onto uncoated bacteriological 35-mm dishes (Coning Inc., Corning, NY, USA) in 3D Tumorsphere Medium XF (Sigma) [39]. After 7 days of culture, images of the spheres were acquired using an inverted microscope coupled with a camera (Carl Zeiss, Göttingen, Germany). The captured images were analyzed on a computer, and the number of spheres was measured using ImageJ software (version 1.52; NIH, Bethesda, MD, USA).

### Fluorescent labeling of LA

To assess the intracellular localization of LA, it was labeled with the fluorescent reagent 9-anthryldiazomethane (ADAM; Funakoshi, Tokyo, Japan). ADAM reacts with fatty acids to form esters that emit fluorescence with strong intensity. Methanol (1 mL) was added to ADAM (1 mg) to prepare a 0.1% ADAM reaction solution. The reaction solution (100 μL) and LA (100 μL) were mixed in the dark at room temperature for 2 h. CT26 cells were then treated with labeled LA (35 μg/mL) and Mitogreen (100 nM)(Molecular Probes, Eugene, OR, USA) for visualizing mitochondria. Images were observed using a fluorescence microscope (BZ-X700, KEYENCE, Osaka, Japan). Fluorescence intensities of the labeled-LA solution (35 μg/mL) and the whole cell lysate and nuclear fraction of labeled LA-treated CT26 cells were measured by a fluorescence spectrophotometer (F-2700, Hitachi High-Tech GLOBAL, Tokyo, Japan).

### RT-PCR

To assess murine mRNA expression, RT-PCR was performed with 2 μg total RNA extracted from CT26 cells using TRI REAGENT (Molecular Research Center, Inc., Cincinnati, OH, USA) according to the manufacturer’s protocol. cDNA was synthesized with 0.5 μg total RNA using a High Capacity cDNA Reverse Transcription Kit (Applied Biosystems, Waltham, MA, USA). The primer sets are listed in Table 1 and were synthesized by Sigma Genosys (Ishikari, Japan). PCR products were electrophoresed on a 2% agarose gel and stained with ethidium bromide. *Gapdh* mRNA was also amplified for use as an internal control.

**Table 1:** Primer pairs used in this study

Gene symbol	Gene bank ID	Forward primer (5′–3′)	Reverse primer (5′–3′)
*Gapdh*	NM_001289726.1	aactttggcattgtggaagg	acacattgggggtaggaaca
*Shh*	NM_009170.3	ctggccagatgttttctggt	gatgtcggggttgtaattgg
*Ptch*	NM_008957.3	ctcaggcaatacgaagcaca	gacaaggagccagagtccag
*Smo*	NM_176996.4	ttgtgctcatcaccttcagc	tgccaaacatggcaaataga
*Gli1*	AB025922.1	tctgtgatgggcaatggtct	tctggggtgggatcaggata
*Gli2*	NM_001081125.1	cagccttcacttttccccac	ctgcttgttctggttggcat
*Gli3*	NM_008130.2	tgcccatcagctactcagtg	ttgttgcagagtgaggttgc
*Sox17*	BC060612.1	gcacagcagaacccagatct	ccggtacttgtagttggggt
*Pax6*	AF443223.1	aggaaccagagaagacaggc	caggttgtttgccatggtga
*Nanog*	AY278951.1	atgcggactgtgttctctca	tgctgagcccttctgaatca
*Col1a*	NM_007742.4	atgtgccactctgactggaa	tccatcggtcatgctctctc

### Quantitative RT-PCR (qRT-PCR)

The extraction of total RNA was carried out using a RNeasy Mini Kit (Qiagen Genomics, Bothell, WA, USA) and cDNA (1 μg) was synthesized with the ReverTra Ace-α-RT Kit (Toyobo, Osaka, Japan). qRT-PCR was performed using the StepOne Real-Time PCR System (Applied Biosystems, Foster City, CA, USA) using the Fast SYBR Green Master Mix (Applied Biosystems) and analyzed using the relative standard curve quantification method [40]. PCR conditions were set according to the manufacturer’s instructions. Actin B (ACTB; GenBank accession No. NM 001101) was used as internal control. Each amplification reaction was evaluated by a melting curve analysis. For the visualization the PCR products, agarose gel electrophoresis and ethidium bromide staining were performed.

### Western blotting

The levels of cleaved GLI2 in the nucleus were measured using western blotting. Whole-cell lysates were prepared as previously described using RIPA buffer supplemented with 0.1% SDS (Thermo Fisher Scientific, Tokyo, Japan) [41]. The Minute Cytoplasmic and Nuclear Extraction Kit (Invent, Biotechnologies, Inc., US) was used to extract nuclear protein. Protein assays were performed using the Protein Assay Rapid Kit (Wako). Lysates were separated using 7.5% or 10.0% SDS-polyacrylamide gel electrophoresis and transferred to nitrocellulose membranes. The membranes were incubated with a primary antibody specific to GLI2 (ab223651, Abcam), or a polyclonal rabbit/mouse anti-IgG antibody (DAKO) at room temperature for 2 hours. β-Actin (anti-β-actin antibody, clone sc47778, Santa Cruz Biotechnology) was used as a loading control. Immune complexes were visualized using Fusion Solo (M&S Instruments, Osaka, Japan). Images were captured on a computer and the signal strength was measured using NIH ImageJ software.

### Immunoprecipitation

Immunoprecipitation was performed according to a previously described method [42, 43]. Lysates were pre-cleaned in lysis buffer with protein A/G agarose (Santa Cruz Biotechnology) for 1 h at 4°C and subsequently centrifuged. The supernatants were then incubated with a precipitation antibody against ubiquitin (Santa Cruz Biotechnology) and protein A/G agarose for 1.5 h at 4°C. Precipitates were collected by centrifugation, washed thrice with wash buffer, and solubilized with 4× Laemmli Sample Buffer (Bio-Rad, Hercules, CA, USA) and 2-mercaptoethanol (Sigma). Finally, immunoblotting was performed using antibodies against GLI2.

### Small interfering RNA

Stealth Select RNAi (siRNA) targeting human and mouse *GLI2* were purchased from Sigma. AllStars Negative Control siRNA was used as a control (Qiagen; Valencia, USA). The cells were transfected with 10 nM siRNA using Lipofectamine 3000 (Thermo Fisher) according to the manufacturer’s recommendations.

### LA binding assay

ADAM-labeled LA (ADAM-LA, 100 μM) was incubated in a 10-μl reaction solution [1 μg acyl-CoA synthetase (Proteintech Group, Inc., Rosemont, IL, USA), 50 mM Tris-Cl (pH 8.0), 5 mM ATP, 2.5 mM MgCl2 and 2 mM coenzyme A. After prewarming the reaction mixture at 30°C for 15 min, mouse recombinant GLI2 (rGLI2, 20 μg, CUSABIO, Huston, TX, USA) was added to a final reaction volume of 20 μl and incubated for 5 min at 30°C [44]. The reactant was transported to CT26 cells by Chariot (Active Motif, Carlsbad, CA, USA). After 12 h culture, whole cell lysate was extracted from CT26 cells to detect GLI2 and cleaved GLI2 by western blot. The western blot membrane soaked with methanol was observed under excitation wavelength 365 nm and fluorescence wavelength 412 nm by a luminoimager (LuminoGraph II, Atto Corp., Tokyo, Japan).

### Animals

Male BALB/c mice and BALB/c Slc-nu/nu mice of four-weeks-old were purchased from SLC Japan, Inc. (Shizuoka, Japan). The animals were maintained in a pathogen-free animal facility under 23°C, 50% humidity, and a 12-h light/12-h dark cycle environment. The animal study was conducted in accordance with the institutional guidelines approved by the Committee for Animal Experimentation of Nara Medical University, Kashihara, Japan, following current regulations and standards of the Japanese Ministry of Health, Labor and Welfare (Approval number 9559, 11365, 11528, 11569 and 11596). Animals were acclimated to their housing for seven days before the start of the experiment. Mice were fed with CE-2 standard diet (CLEA Japan, Inc., Tokyo, Japan) or experimental diet prepared by mixing LA (Wako Pure Chemical Industries, Ltd., Osaka, Japan) into CE-2 diet by 15% w/w concentration [45].

### Orthotopic liver metastasis model

For the establishment of liver metastasis models, CT26 and HT29 cancer cells (1 × 10^6^ in 40 μL phosphate-buffered saline) were inoculated into the cecal submucosal layer of syngeneic BALB/c mice (*n* = 60) and BALB/c Slc-nu/nu mice (*n* = 60), respectively [46]. The two kinds of mice were randomly divided into three groups: CE-2 diet group (*n* = 20), LA diet group (*n* = 20), and *GLI2* knockdown + LA diet group (*n* = 20), respectively. In the GLI2 knockdown + LA diet group group, cancer cells knocked down *GLI2* with *GLI2* siRNA prior to inoculation. The LA diet was given only for the first week and then the CE-2 diet. Five mice in each group were euthanized weekly and the size of the primary tumor and the number of liver metastases were measured. The excised livers were sectioned into 2-mm-thick slices, and metastatic foci were counted using a stereomicroscope (Nikon) [47]. For examination of cleaved GLI2 fragment, tumors of the mice euthanized after two weeks from the inoculation were subjected for immunoblotting.

### Plasma LA concentration

The plasma LA concentration was analyzed by gaschromatography Total lipids were extracted by the methods of Folch et al. [48]. The fatty acids in total lipids were transmethylated with 6% sulfuric acid in anhydrous methanol and analyzed on a C-14 gas-chromatograph (Shimazu, Kyoto, Japan) equipped with an SP-2330 capillary column (Supelco, Bellefonte, PA, USA).

### Histological analysis

The formalin-fixed tissues were dehydrated and embedded in paraffin. After slicing the created block to 3 μm, hematoxylin and eosin staining was performed to observe the morphology.

### Patients

We obtained frozen tissue samples from 11 patients with CRC with serosal invasion (pT3) and 1–3 regional lymph node metastasis (pN1), which were diagnosed at the Department of Molecular Pathology, Nara Medical University, during 2012–2019. Four cases were no distant metastasis; seven cases showed liver metastasis. As written informed consent was not obtained from the patients for their participation in the present study, all identifying information was removed from patient samples prior to their analysis to ensure strict privacy protection (unlinkable anonymization). All procedures were performed in accordance with the Ethical Guidelines for Human Genome/Gene Research enacted by the Japanese Government and with the approval of the Ethics Committee of Nara Medical University (approval number: 937, 2012/4/1).

### Statistical analysis

Statistical significance was calculated using a two-tailed Fisher’s exact test and ordinary ANOVA using the InStat software (GraphPad, Los Angeles, CA, USA). A two-sided *P* value of <0.05 was considered to indicate statistical significance.

## Abbreviations

LA: linoleic acid
CRC: colorectal cancer
SHH: sonic hedgehog
GLI: glioma-associated oncogene homolog
SOX: SRY-box transcription factor
PTCH: patched
SMO: smoothened
PKA: protein kinase A
cAMP: cyclic adenosine monophosphate
